# Influence of VO_2_ Nanoparticle Morphology on the Colorimetric Assay of H_2_O_2_ and Glucose

**DOI:** 10.3390/nano7110347

**Published:** 2017-10-25

**Authors:** Rui Tian, Jiaheng Sun, Yanfei Qi, Boyu Zhang, Shuanli Guo, Mingming Zhao

**Affiliations:** School of Public Health, Jilin University, Changchun 130021, Jilin, China; tianrui16@mails.jlu.edu.cn (R.T.); sunjh15@mails.jlu.edu.cn (J.S.); boyu17@mails.jlu.edu.cn (B.Z.); guosl15@mails.jlu.edu.cn (S.G.); mingming17@mails.jlu.edu.cn (M.Z.)

**Keywords:** VO_2_ nanoparticles, morphology, nanozyme, colorimetric sensor

## Abstract

Nanozyme-based colorimetric sensors have received considerable attention due to their unique properties. The size, shape, and surface chemistry of these nanozymes could dramatically influence their sensing behaviors. Herein, a comparative study of VO_2_ nanoparticles with different morphologies (nanofibers, nanosheets, and nanorods) was conducted and applied to the sensitive colorimetric detection of H_2_O_2_ and glucose. The peroxidase-like activities and mechanisms of VO_2_ nanoparticles were analyzed. Among the VO_2_ nanoparticles, VO_2_ nanofibers exhibited the best peroxidase-like activity. Finally, a comparative quantitative detections of H_2_O_2_ and glucose were done on fiber, sheet, and rod nanoparticles. Under the optimal reaction conditions, the lower limit of detection (LOD) of the VO_2_ nanofibers, nanosheets, and nanorods for H_2_O_2_ are found to be 0.018, 0.266, and 0.41 mM, respectively. The VO_2_ nanofibers, nanosheets, and nanorods show the linear response for H_2_O_2_ from 0.025–10, 0.488–62.5, and 0.488–15.625 mM, respectively. The lower limit of detection (LOD) of the VO_2_ nanofibers, nanosheets, and nanorods for glucose are found to be 0.009, 0.348, and 0.437 mM, respectively. The VO_2_ nanofibers, nanosheets, and nanorods show the linear response for glucose from 0.01–10, 0.625–15, and 0.625–10 mM, respectively. The proposed work will contribute to the nanozyme-based colorimetric assay.

## 1. Introduction

Natural enzymes with great catalytic capacity and high substrate specificity have attracted much research interest in the fields of medicine, biology, and food industry. Despite these broad developments, natural enzymes often have inherent drawbacks, such as high preparation and purification costs, low operational stability, sensitivity of catalytic activity to environmental conditions, and difficulty of recovery. These shortcomings are limited to its practical application [1]. Artificial mimic enzymes have the characteristics of high catalytic efficiency, stability, economy, and large-scale preparation which has been rapidly developed in the fields of medicine, chemical industry, food, agriculture, environmental science, and analytical chemistry [2]. Among the various artificial mimic enzymes, nanozymes as the new-generation emzyme-mimetic have attracted considerable interest since the ferroferric oxide nanomaterial has the catalytic properties similar to horseradish peroxidase (HRP) [3]. Many nanoparticles have been studied as enzyme mimetics, including ferromagnetic NPs [3,4,5,6,7,8,9,10,11], cerium oxide NP [12,13,14], metal NPs [15,16,17,18,19,20,21,22,23], carbon-based nanomaterials [24,25,26,27,28], V_2_O_5_ nanowires [29,30], and perovskite oxide [31,32].

Vanadium dioxide (VO_2_) have received considerable attention for their redox activity and layered structures, which can serve as very good intercalation materials and smart sensors [33]. The VO_2_ exists in multiple morphologies, such as fibers, nanorods, nanosheets, spheres, and hollow spheres [34,35]. The shape of the nanoparticle has attracted growing interest due to its effect on the catalytic, optical, electronic, and magnetic properties [9,36,37,38,39,40,41]. For example, one-dimensional (1D) nanostructures—such as nanotubes, nanorods, and nanowires—exhibit higher activity and durability, compared with zero-dimensional (0D) nanostructures, due to possessing fewer lattice boundaries, fewer defect sites, and longer segments of surface crystalline planes [36]. Therefore, we focused on the effect of different morphology on the catalytic activities of VO_2_ nanoparticles in order to obtain more information for their potential applications in biosensor and biocatalysts. 

Herein, different morphologies VO_2_ nanoparticles—including fibers, sheets, and rods—were synthesized. The catalysis activities and kinetic mechanic of various VO_2_ nanoparticles were investigated upon the reaction of hydrogen peroxide with its reducing substrates 3,3′,5,5′-tetramethybenzidine (TMB). The hydrogen peroxide and glucose colorimetric sensors were developed based on VO_2_ nanoparticles with different shapes. In this colorimetric assay, different analytical parameters—such as concentrations of nanoparticles, buffer solution, and pH of the analyte medium—were determined. Under optimal reaction conditions, the detection system of fiber-like VO_2_ nanoparticles shows the most sensitive response to H_2_O_2_ and glucose than the other two VO_2_ nanoparticles.

## 2. Results and Discussions

### 2.1. Characterization of VO_2_ Nanoparticles

**Figure 1:**
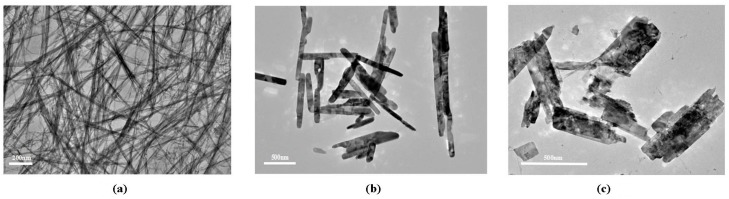
TEM images of VO_2_ nanoparticles. (**a**) VO_2_ nanofibers (**b**) VO_2_ nanosheets (**c**) VO_2_ nanorods.

**Figure 2:**
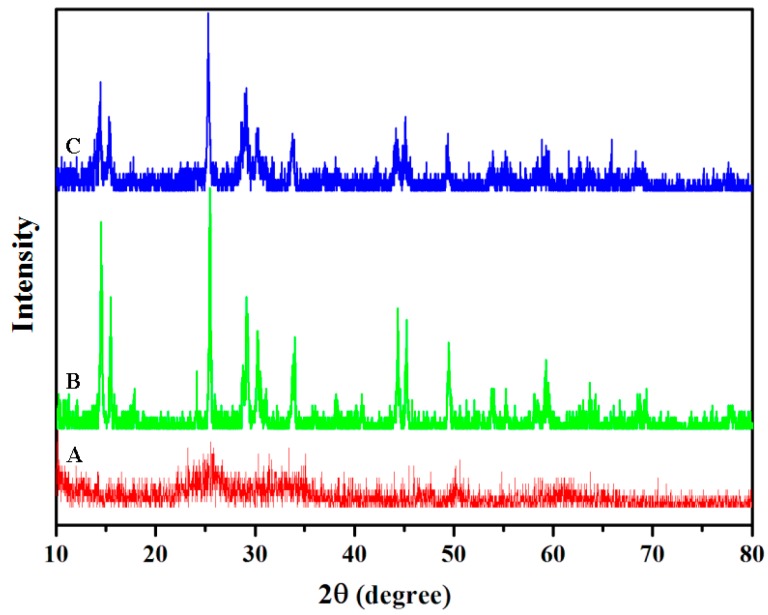
XRD patterns of VO_2_ nanoparticles. (**A**) VO_2_ nanofiber; (**B**) VO_2_ nanosheets; (**C**) VO_2_ nanorods.

The structural characterizations of the VO_2_ nanoparticles were done by transmission electron microscopy (TEM) and X-ray powder Diffraction (XRD). TEM images indicate the VO_2_ nanoparticles of different morphology, fibers, rods, and sheets (Figure 1). The formation of VO_2_ nanoparticles is confirmed from the X-ray diffraction pattern (Figure 2). The VO_2_ nanoparticles with fiber, sheet, and rod shapes have the same crystal structures as those reported in the literature [34,35], and are monoclinic VO_2_ (Joint Committee on Powder Diffraction Standards card No. 31-1438 and No. 65-7960: see Figure 2).

### 2.2. Principle

**Figure 3:**
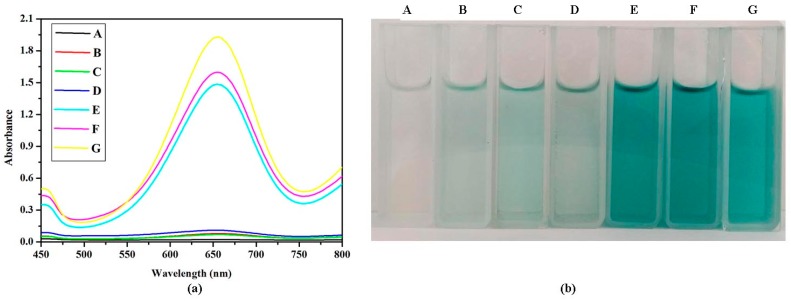
UV-Visible absorption spectra (**a**) and color changes (**b**) of different reaction systems. ((**A**) TMB + H_2_O_2_, (**B**) TMB + VO_2_ nanorod, (**C**) TMB + VO_2_ nanosheet, (**D**) TMB + VO_2_ nanofiber, (**E**) TMB + VO_2_ nanorod + H_2_O_2_, (**F**) TMB + VO_2_ nanosheet + H_2_O_2_, (**G**) TMB + VO_2_ nanofiber + H_2_O_2_).

In pH 4 citrate buffer solution at room temperature, VO_2_ nanoparticles with different morphologies catalyzed the oxidation of a peroxidase substrate 3,3′,5,5′-tetramethylbenzidine (TMB) in the presence of H_2_O_2_ to obtain the TMB oxidized product with blue color. As shown in Figure 3, when various VO_2_ nanoparticles were added into the TMB/H_2_O_2_ solution, the strong absorption peaks were obtained at 656 nm. However, there were no strong absorption peaks when the solution did not contain H_2_O_2_ or VO_2_ nanoparticles. The absorbance becomes stronger due to more TMB being oxidized with the increasing of the concentration of H_2_O_2_. The absorbance also showed a linear trend depending on the concentration of H_2_O_2_. 

### 2.3. Effect of pH

**Figure 4:**
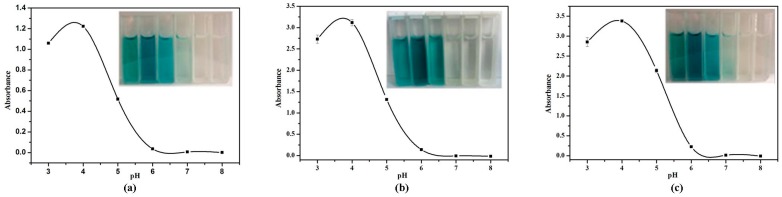
The effect of pH on absorption value with TMB and color changes. (**a**) VO_2_ nanofibers; (**b**) VO_2_ nanosheets; (**c**) VO_2_ nanorods. The error bars represent the standard deviation of three measurements.

The effect of pH value (pH 3.0–8.0) on absorption value with TMB was investigated in the citrate buffer system, as shown in Figure 4. Each of the VO_2_ nanofibers, nanosheets, and nanorods of the system reached their maximum peaks when the pH value was 4.0. Therefore, pH 4.0 was selected to detect H_2_O_2_ and glucose with various VO_2_ nanoparticles.

### 2.4. Effect of Buffers

**Figure 5:**
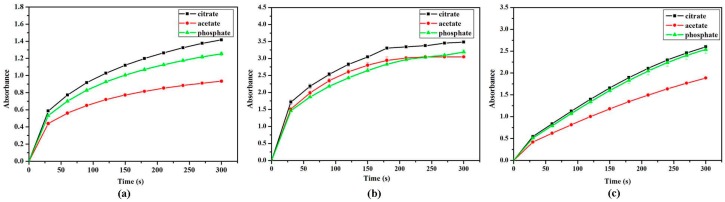
The effect of buffer solution on absorption value with TMB. (**a**) VO_2_ nanofibers; (**b**) VO_2_ nanosheets; (**c**) VO_2_ nanorods. The error bars represent the standard deviation of three measurements.

The effect of buffers on absorption value of TMB oxide product was examined. The time response curves of TMB with H_2_O_2_ catalyzed by VO_2_ with different morphologies, in pH 4.0, 0.2 M acetate, phosphate, and citrate buffers. The results were shown in Figure 5. Up to 300 s, the VO_2_ nanoparticles were more active in the citrate buffer solution. Thus, the citrate buffer solution (pH = 4.0, 0.2 M), was chosen as the optimal reaction solution for the H_2_O_2_ and glucose colorimetric assay.

### 2.5. Effect of VO_2_ Nanoparticle Morphologies and Concentrations

**Figure 6:**
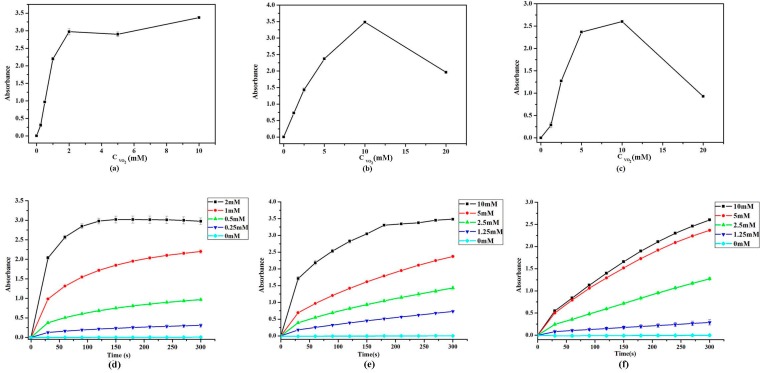
The effect of VO_2_ nanofibers (**a**,**d**); VO_2_ nanosheets (**b**,**e**); and VO_2_ nanorods (**c**,**f**) concentrations on absorption value with TMB in pH 4.0 citrate buffer solution. The error bars represent the standard deviation of three measurements.

As shown in Figure 6, the absorption values at OD_656nm_ of TMB oxide product increased gradually with the concentration of VO_2_ nanoparticles. The system reached its maximum absorption value when the concentrations of VO_2_ nanofibers, nanosheets, and nanorods were 10, 10, and 2 mM, respectively. The results show that the catalytic activity of VO_2_ nanofibers is stronger than the other two, shown in the Figure 6.

### 2.6. Steady-State Kinetic Assay

**Figure 7:**
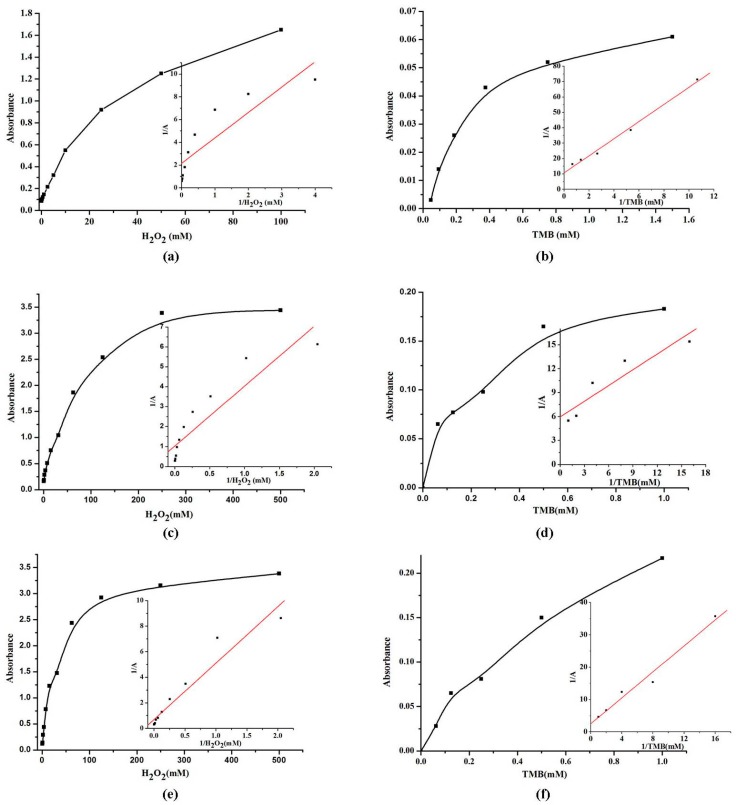
The steady-state kinetic assay and catalytic mechanism of VO_2_ nanofibers (**a**,**b**); VO_2_ nanosheets (**c**,**d**); and VO_2_ nanorods (**e**,**f**) as peroxidase mimics. Conditions: pH, 4.0 (0.2 M citrate buffer); temperature, 25 °C; incubation time, 5 min.

For further understanding the influence of particle morphology on the catalytic mechanism of VO_2_ nanoparticles, the steady-state kinetic assay for VO_2_ nanoparticles were determined in detail. As shown in Figure 7, the typical Michaelis-Menten curve were obtained for VO_2_ nanozymes. Michaelis-Menten constant (K_M_) and maximum initial velocity (V_max_) were known from Michaelis-Menten curve use a Lineweaver-Burk plot. A comparison of the kinetic parameters of VO_2_ nanozymes, V_2_O_5_ nanozymes, Fe_3_O_4_ magnetic nanoparticle (MNP_S_), and HRP was given in Table 1. The K_M_ of VO_2_ nanofibers, nanosheets, and nanorods with TMB were 0.518, 0.111, and 0.801 mM, respectively. The V_max_ of VO_2_ nanofibers, nanosheets, and nanorods with TMB were 9.3 × 10^−5^, 1.68 × 10^−4^, and 3.99 × 10^−4^ M·s^−1^, respectively. The K_M_ of VO_2_ nanofibers, nanosheets, and nanorods with H_2_O_2_ were 1.043, 2.924, and 6.469 mM. The V_max_ of VO_2_ nanofibers, nanosheets, and nanorods with H_2_O_2_ were 4.66 × 10^−4^, 9.73 × 10^−4^, and 1.46 × 10^−3^ M·s^−1^, respectively. The K_M_ values shows that VO_2_ nanosheets with TMB as the substrate was apparently lower than VO_2_ nanofibers, VO_2_ nanorods, V_2_O_5_ nanozymes, Fe_3_O_4_ MNP_S_, and HRP. It shows that the VO_2_ nanosheets have a higher affinity to TMB compared with VO_2_ nanofibers, VO_2_ nanorods, Fe_3_O_4_ MNP_S_, and HRP. Which means that a lower TMB concentration was required to reach the maximal activity for VO_2_ nanosheets. The apparent K_M_ values of VO_2_ nanofibers with H_2_O_2_ as the substrate was apparently lower than VO_2_ nanorods, VO_2_ nanosheets, Fe_3_O_4_ MNP_S_, and HRP. It shows that the VO_2_ nanofibers have a higher affinity for H_2_O_2_ compared with VO_2_ nanosheets, VO_2_ nanorods, Fe_3_O_4_ MNP_S_, and HRP. That means a lower H_2_O_2_ concentration was required to reach the maximal activity for VO_2_ nanofibers. 

### 2.7. Calibration Curve for H_2_O_2_ and Glucose Detection

**Figure 8:**
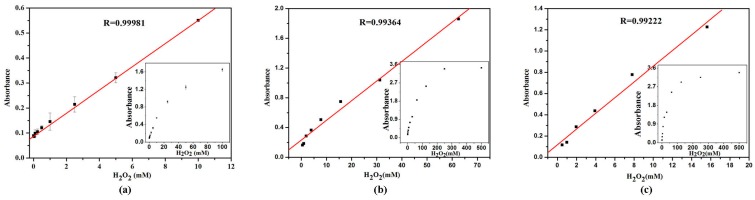
A dose-response curve depending of the absorbance at 656 nm in the presence of different concentrations of H_2_O_2_. (**a**) VO_2_ nanofibers; (**b**) VO_2_ nanosheets; (**c**) VO_2_ nanorods. Error bars represent the standard deviation of three measurements. Conditions: pH, 4.0 (0.2 M citrate buffer); temperature, 25 °C; incubation time, 5 min.

Under the optimal conditions (pH 4.0 citrate buffer, the concentrations of VO_2_ nanofibers, nanosheets and nanorods were 2, 10, and 10mM, respectively.) the calibration curves of H_2_O_2_ were obtained with VO_2_ nanoparticles different morphologies (Figure 8). The correlation between the absorbance values and H_2_O_2_ concentration are linear over the range of 0–100 mM (nanofibers), 0–500 mM (nanosheets) and 0–500 mM (nanorods) with correlation coefficients 0.99981, 0.99364, and 0.99222, respectively. The lower limit of detection (LOD) of the VO_2_ nanofibers, nanosheets, and nanorods for H_2_O_2_ are found to be 0.018, 0.266, and 0.41 mM, respectively. 

**Figure 9:**
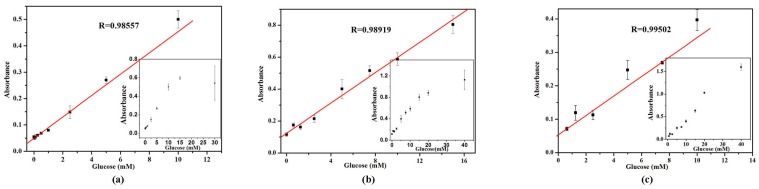
A dose-response curve depending of the absorbance at 656 nm in the presence of different concentrations of glucose. (**a**) VO_2_ nanofibers; (**b**) VO_2_ nanosheets; (**c**) VO_2_ nanorods, in which error bars represent the standard deviation of three measurements. Conditions: pH, 4.0 (0.2 M citrate buffer); temperature, 25 °C; incubation time, 5 min.

As glucose oxidase (GOx) can catalyze the oxidation of glucose and produce H_2_O_2_, the absorption value with TMB was changing by H_2_O_2_ in presence of VO_2_ nanoparticles. Because the GOx would be denatured in pH 4.0 buffer, the glucose detection was produced in two steps: first, H_2_O_2_ was induced by GOx oxidation of glucose and then the reaction solutions were detected by TMB/different VO_2_ nanoparticles system. As shown in Figure 9, the absorbtion increases gradually with the increasing of glucose concentration. The correlation between the absorbance at 656 nm and glucose concentration are linear over the range of 0–30 mM (nanofibers), 0–40 mM (nanosheets) and 0–40 mM (nanorods) with the correlation coefficient of 0.98557, 0.98919, and 0.99502, respectively. The lower limit of detection (LOD) of the VO_2_ nanofibers, nanosheets, and nanorods for glucose are found to be 0.009, 0.348, and 0.437 mM, respectively. 

The VO_2_ nanofibers showed the highest peroxidase activity in the H_2_O_2_ and glucose colorimetric assay, followed by VO_2_ nanosheets, and finally VO_2_ nanorods. Additionally, it was reported that the specific surface area of VO_2_ nanoparticles greatly influences their catalytic activities. The specific surface area of VO_2_ nanofibers (185 to 122 m^2^ g^−1^) [34] is also much larger than that of other VO_2_ micro/nanoparticles, such as hollow microspheres (22.3 m^2^ g^−1^), nanowires (12.3 m^2^ g^−1^) [42], nanobelts (18.6 m^2^ g^−1^) [43], nanorods (42 m^2^ g^−1^) [44], VO_2_ mesocrystals (28.4 m^2^ g^−1^) [45], mesoporous VO_2_ nanowires (46.7 m^2^ g^−1^) [46], and 3D GO-VO_2_ nanosheet flowers (71.6 m^2^ g^−1^) [47]. Therefore, the VO_2_ nanofibers demonstrated the most sensitive response during the H_2_O_2_ and glucose sensing. By comparing with other nanozymes to further understanding the catalytic activity of VO_2_ nanozymes as peroxidase mimetics, as shown in Table 2, the VO_2_ nanofibers have a wider linear range. 

## 3. Materials and Methods 

### 3.1. Chemicals and Materials

All the chemicals used were of analysis grade without further purification. 3,3′,5,5′-Tetramethylbenzidine (TMB) was obtained from Tokyo Chemical Industry Co., Ltd. (Tokyo, Japan). Glucose oxidase (GOx) was obtained from Aladdin Reagent Co., Ltd. (Shanghai, China). V_2_O_5_, oxalic acid, methanol, glucose, hydrogen peroxide (H_2_O_2_, 30%), etc., were purchased from Beijing Chemical Works (Beijing, China). The water used in the experiments was purified.

### 3.2. Synthesis of VO_2_ Nanoparticles

The synthesis of VO_2_ nanofiber contains two steps: synthesis of VO_2_ hollow sphere and the supernatant collecting and drying. According to the literature procedure [35] synthesis of VO_2_ hollow sphere, with minor adjustment. Briefly, V_2_O_5_ and oxalic acid (the ratio of molar is 1:3) were first dissolved in 7 mL distilled water and stirred for 10 min at room temperature. Then the 23 mL methanol was added in the solution and stirred for another 10 min. The mix solution was transferred to a Teflon-lined autoclave with stainless steel, and heated at 200 °C for 24 h. The sample was cooled down naturally. The black precipitates were filtered off and washed with distilled water and ethanol, and then dried at 80 °C overnight, and finally the VO_2_ hollow spheres were dissolved, the supernatant was collected and dried. Similar procedures were adopted to prepare nanorods and nanosheets: when the water content is 10 mL, the product is nanorods, and when the solution is completely water, the product is just nanosheets (with water and methanol measures maintained at 30 mL).

### 3.3. Physical Characterization

The morphology and size of the VO_2_ nanoparticles were acquired using a transmission electron microscopy (TEM) by JEM-1011 transmission electron microscopy (JEOL, Tokyo, Japan) with a working voltage at 100 kV. The X-ray powder diffraction method was carried out in a D/max-rα power diffractometer (Rigaku, Tokyo, Japan) using Cu-Kα monochromatic radiation (λ = 1.5418 Å).

### 3.4. H_2_O_2_ Detection Using VO_2_ Nanoparticles as Peroxidase Mimetics

To discover the peroxidase-like character of VO_2_ nanoparticles, the experiments were performed as follows: 60 μL VO_2_ nanoparticles solution (the concentrations of nanofibers, nanosheets, and nanorods are 2, 10, and 10 mM, respectively) in a reaction volume of 2400 μL citrate buffer solution (pH = 4.0) and 480 μL TMB solution (1.5 mM in ethanol), followed by the addition of 60 μL H_2_O_2_ (30%). The mixed solution was reacted for 5 min at room temperature. Then used for the UV-Vis spectrophotometer (Metash Instruments Inc., Shanghai, China) record the spectra at 656 nm for TMB.

To investigate the influence of buffer solution on the VO_2_ nanoparticle characteristics, the pH—ranging from 3.0 to 8.0 of the buffer solution—was examined, under conditions identical to these used above.

To investigate the influence of different reaction buffers on the VO_2_ nanoparticles characteristics, catalytic reactions incubated in difference buffer solution—including citrate, phosphate, and acetate—were examined, under conditions identical to these used in above. For a blank, only substrate solution was used. All experiments were conducted at room temperature (25 °C).

### 3.5. Glucose Detection Using VO_2_ Nanoparticles

Glucose detection was examined as follows: (a) 200 μL of GOx (1 mg/mL) and 200 μL of glucose of different concentrations in 400 μL of phosphate buffered saline (PBS, pH = 7.0) were incubated at 37 °C for 60 min; (b) 400 μL of TMB (1.5 mM in ethanol) and 50 μL of VO_2_ nanoparticles solution (the concentrations of nanofibers, nanosheets, and nanorods are 2, 10, and 10 mM, respectively) in 1750 μL of citrate buffer solution (pH = 4.0) were added into the above glucose reaction solution; (c) The mixed solutions with different concentrations of glucose were incubated for 5 min; the (d) the UV-Vis spectrophotometer was used to record the spectra. 

## 4. Conclusions

VO_2_ nanoparticles with different structures—nanofibers, nanosheets, and nanorods—have been successfully fabricated and show peroxidase-like activities. The catalytic behaviors of VO_2_ nanoparticles show Michaelis-Menten kinetics and good affinity to both H_2_O_2_ and TMB. The VO_2_ nanoparticle-based colorimetric assay provides fast, sensitive, and low-cost H_2_O_2_ and glucose sensors. Compared with VO_2_ nanorods and VO_2_ nanosheets, the VO_2_ nanofibers demonstrated the most sensitive response during the H_2_O_2_ and glucose sensing. This investigation is significant for vanadium-based nanozyme application in biosensor and biocatalysis.

## Figures and Tables

**Table 1:** Comparison of the K_M_ and V_max_ of VO_2_ nanozymes, V_2_O_5_ nanozymes, Fe_3_O_4_ MNP_S_, and HRP, respectively.

Nanozymes	Substrate	K_M_ (mM)	V_max_ (M·S^−1^)
VO_2_ nanofibers	TMB	0.518	9.3 × 10^−5^
VO_2_ nanofibers	H_2_O_2_	1.043	4.66 × 10^−4^
VO_2_ nanosheets	TMB	0.111	1.68 × 10^−4^
VO_2_ nanosheets	H2O2	2.924	9.73 × 10^−4^
VO_2_ nanorods	TMB	0.801	3.99 × 10^−4^
VO_2_ nanorods	H_2_O_2_	6.469	1.46 × 10^−3^
V_2_O_5_ nanozymes	TMB	0.738	1.85 × 10^−5^
V_2_O_5_ nanozymes	H_2_O_2_	0.232	1.29 × 10^−5^
Fe_3_O_4_ MNPS	TMB	0.434	10.00 × 10^−8^
Fe_3_O_4_ MNPS	H_2_O_2_	154	9.78 × 10^−8^
HRP	TMB	0.434	1.24 × 10^−8^
HRP	H_2_O_2_	3.70	2.46 × 10^−8^

**Table 2:** Comparison of different nanozymes for the detection of H_2_O_2_.

Nanozymes	Linear Range	Limit of Detection	Reference
Fe_3_O_4_ MNP_S_	1–100 μΜ	0.5 μΜ	[48]
HRP	1–60 μΜ	1 μΜ	[49]
Pt-DNA complexes	0.979–17.6 mΜ	0.392 mΜ	[50]
V_2_O_5_ nanozymes	1–500 μΜ	1 μΜ	[30]
VO_2_ nanofibers	0.025–10 mM	0.018 mM	This work
VO_2_ nanosheets	0.488–62.5 mM	0.266 mΜ	This work
VO_2_ nanorods	0.488–15.6 mM	0.41 mΜ	This work
